# Combined Anti-Cancer Effects of Platycodin D and Sorafenib on Androgen-Independent and PTEN-Deficient Prostate Cancer

**DOI:** 10.3389/fonc.2021.648985

**Published:** 2021-05-07

**Authors:** Zongliang Lu, Wei Song, Yaowen Zhang, Changpeng Wu, Mingxing Zhu, He Wang, Na Li, Yong Zhou, Hongxia Xu

**Affiliations:** ^1^ Department of Clinical Nutrition, Daping Hospital, Army Medical University, Chongqing, China; ^2^ Department of Clinical Nutrition, Banan District People’s Hospital of Chongqing, Chongqing, China

**Keywords:** Platycodin D, Sorafenib, PTEN, FOXO3a, Akt

## Abstract

Castration-resistant (androgen-independent) and PTEN-deficient prostate cancer is a challenge in clinical practice. Sorafenib has been recommended for the treatment of this type of cancer, but is associated with several adverse effects. Platycodin D (PD) is a triterpene saponin with demonstrated anti-cancer effects and a good safety profile. Previous studies have indicated that PC3 cells (PTEN -/-, AR -/-) are sensitive to PD, suggesting that it may also be a useful treatment for castration-resistance prostate cancer. We herein investigated the effects of combining PD with sorafenib to treat PTEN-deficient prostate cancer cells. Our data show that PD promotes sorafenib-induced apoptosis and cell cycle arrest in PC3 cells. Of interest, PD only promoted the anti-cancer effects of sorafenib in Akt-positive and PTEN-negative prostate cancer cells. Mechanistic studies revealed that PD promoted p-Akt ubiquitination by increasing the p-Akt level. PD also increased the protein and mRNA expression of FOXO3a, the downstream target of Akt. Meanwhile, PD promoted the activity of FOXO3a and increased the protein expression of Fasl, Bim and TRAIL. Interestingly, when FOXO3a expression was inhibited, the antitumor effects of both PD and sorafenib were individually inhibited, and the more potent effects of the combination treatment were inhibited. Thus, the combination of PD and sorafenib may exert potent anti-cancer effects specifically *via* FOXO3a. The use of Akt inhibitors or FOXO3a agonists, such as PD, may represent a promising approach for the treatment of androgen-independent and PTEN-deficient prostate cancer.

## Introduction

According to 2019 data, the incidence and death rates of prostate cancer are first and second in men in the USA (1). Androgen deprivation therapy (ADT) is the main treatment for early prostate cancer, and most patients are initially sensitive to ADT. However, the intrinsic heterogeneity of tumor cells and the development of gene mutations driven by therapeutic drugs can result in resistance to ADT and progression to castration-resistant prostate cancer (CRPC) (2). Although chemotherapeutic drugs such as docetaxel, enzalutamide and abiolone prolong the survival of patients with CRPC, there are differences in their efficacy among patients, and none of the existing treatments is optimal. High-throughput gene sequencing has revealed that there are many gene mutations and the activation of various signaling pathways during the progression from high-grade epithelioid tumors to CRPC, indicating why the responses to targeted therapeutic drugs vary so widely (3, 4). Researchers have proposed a new individualized treatment model for prostate cancer based on gene mutations (4, 5).

PTEN, a tumor suppressor gene, is related to cancer progression and malignancy, and its loss is associated with a poor prognosis (6–9). Approximately half of CRPC cases have mutant PTEN (10). Preclinical data indicated that restoration of PTEN expression inhibited the growth of PTEN-deficient prostate cancer cells (11). As a phosphorylase, PTEN negatively regulates the PI3K/Akt pathway (12). Activation of the PI3K/Akt pathway and androgen receptor (AR) generates reciprocal feedback in PTEN-deficient prostate cancer (13). Clinical data implied that combined blockade with abiraterone (an AR inhibitor) and ipatasertib (an Akt inhibitor) showed superior antitumor activity to abiraterone alone in patients with PTEN-deficient tumors (14). However, sustained AR inhibition leads to a transition from androgen-dependent to independent disease. Of note, restoration of PTEN expression improves the sensitivity of androgen-independent prostate cancer to docetaxel (15). There is currently no effective strategy to treat patients with both androgen-independent and PTEN-deficient prostate cancer.

Sorafenib is a molecular targeted drug approved for the treatment of several human cancers. Steinbild et al. showed that sorafenib is beneficial for patients with progressive hormone-refractory prostate cancer (16). However, research has demonstrated that, besides targeting Raf and vascular endothelial growth factor receptor (VEGFR), sorafenib also inhibits the AR and Akt in prostate cancer (17). Clinical evidence suggests that sorafenib, alone or in combination with bicalutamide (an AR inhibitor), can overcome the resistance of CRPC to chemotherapy (18, 19). Ongoing studies are exploring the response of various phenotypes of prostate cancer to sorafenib. Yamamoto et al. showed that sorafenib decreased the activation of the PI3K/AKT/mTOR signaling axis in a mouse model of PTEN-deficient and castration-resistant prostate cancer, but there was no statistically significant reduction in the tumor burden. Further analysis indicated that sorafenib significantly inhibited cancer cell proliferation, but had minimal effects on the induction of apoptosis (20), supporting the *in vivo* observations.

FOXO3a is a downstream target of the PI3K/Akt axis, and is a known suppressor of primary tumor growth *via* its transcriptional regulation of key genes involved in cell cycle arrest, apoptosis, DNA damage and drug resistance (21–24). Reducing FOXO3a expression accelerates prostate cancer progression (25), while increasing FOXO3a reduces the viability of prostate cancer stem cells (26). Akt phosphorylates FOXO3a at multiple sites and leads to its transportation out of the nucleus and retention in the cytoplasm (27, 28). FOXO3a regulates the protein expression of PTEN, and expression of the PTEN/FOXO3a/PLZF signaling pathway is associated with tumor progression (29). Docetaxel has been shown to induce cancer cell apoptosis at least partly by enhancing FOXO3a activity and stimulating its nuclear translocation (30). The overexpression of FOXO-responsive genes, such as Bim (31), FasL (32) and TRAIL (33), leads to the induction of cell death. However, the response of FOXO3a to sorafenib exposure in prostate cancer cells is unclear. It has been hypothesized that combining conventional treatments with FOXO3a agonists may induce the expression of FOXO3a-related death genes, and may thus increase the anti-tumor effects of sorafenib in PTEN-deficient and androgen independent prostate cancer.

There are currently no specific agonists of FOXO3a. Natural products have been demonstrated to have effects on a variety of transcription factors, and are good choices for treatment due to their diversity, efficacy and safety. Saponins are present in many herbs, and several have been demonstrated to have inhibitory activity against human cancer cell lines, animal models, and have even been evaluated in some clinical trials (34, 35). Platycodin D (PD) is the major triterpene saponin found in the roots of *Platycodon grandiflorum*, which is known as Jiegeng in China. Previous studies have shown that PD inhibits tumor growth and promotes the activity of anti-cancer drugs (36). Our previous studies indicated that PD induces apoptosis and inhibits the cell cycle by regulating FOXO3a and MDM2 in prostate cancer and breast cancer (37, 38). However, the specific mechanism(s) by which FOXO3a is induced by PD to induce cell death are not clear. In addition, it is unclear whether the combination of PD with sorafenib can provide a therapeutic advantage, and if so, the mechanism(s) responsible for the benefits.

To investigate these topics, we herein investigated the impact of and mechanism(s) of action for PD, alone and in combination sorafenib, in PTEN-deficient and androgen-independent prostate cancer.

## Materials and Methods

### Test Compound, Chemicals and Other Reagents

PD was purchased from Must Bio-Technology Co., Ltd. (Chengdu, China) and sorafenib was purchased from Selleckchem (Houston, TX, USA). Fetal bovine serum (FBS) was purchased from Bioind (Biological Industries, BeitHaEmek, Israel). The anti-FOXO3a, anti-FasL, anti-Bim, anti-TRAIL, anti-Akt and anti-Ubiquitin antibodies were purchased from Cell Signaling Technology, Inc. (Danvers, MA, USA). The anti-p-Akt, anti-ERK and anti-p-ERK antibodies were obtained from Abcam (Shanghai, China). The anti-Caspase 3, anti-Cleaved Caspase 3, anti-Caspase 3 and anti-PARP antibodies were obtained from Beyotime Biotechnology, Inc. (Shanghai, China). The anti-CDK4, anti-CDK6 and anti-cyclin D antibodies were obtained from Boster Biological Technology, Inc. (Wuhan, China). The Z-VAD-FMK, MG132, pictilisib and MK2206 were purchased from Selleckchem (Houston, TX, USA). Lipofectamine™ 2000 was purchased from Invitrogen (Shanghai, China). The shRNA-FOXO3a plasmid, cDNA-Akt plasmid and cDNA-FOXO3a were purchased Shanghai GeneChem Co., Ltd (Shanghai, China).

### Cell Lines and Cell Culture

The PC3 (PTEN-deficient) and DU145 (PTEN wild type) cell lines were purchased from the Chinese Academy of Sciences (Shanghai, China). The cell culture procedures have been described in our previous papers (39). In brief, the PC3 and DU145 cell lines were cultured in specific media supplemented with 10% FBS and various growth factors. Third-passage cells were used in all of the experiments.

### Viral and Plasmid Transduction

Lentiviruses encoding shRNA against PTEN and FOXO3a, cDNA for PTEN and Akt, and plasmids for Akt and FOXO3a, were constructed by Shanghai GeneChem Co., Ltd. (Shanghai, China). Empty lentivirus and plasmid constructs were used as the negative controls. Cells were cultured in T25 flasks or 6-well plates at 6*10^5^ or 8*10^4^ cells. Cells were transduced with the shRNA, cDNA, empty lentivirus or empty plasmid at a multiplicity of infection in accordance with the manufacturer’s instructions. Puromycin or hygromycin B were used to select stably-transfected cells during viral transduction. Western blotting was carried out to verify the knockdown efficiency.

### Cell Survival, FITC/PI, Cell Cycle, Ultrastructural and Immunofluorescence Assays

Cell survival (40), FITC/PI and PI (41), cell cycle (42), ultrastructural (43), and immunofluorescence (42) assays were performed according to previously reported procedures. The effects of the compounds on PC3 cell growth were determined using the CCK-8 assay. The effects of the compounds on PC3 cell apoptosis and cell death were examined using a FITC/PI and PI detection kit from BestBio (Shanghai, China). PC3 cells were treated with 2 mM thymidine for 24 h to induce cell cycle synchronization. Synchronized or unsynchronized cells were treated with the compounds at the indicated concentrations for 24 h or 48 h. The effects of the compounds on the PC3 cell morphology were determined using transmission electron microscopy (TEM) or scanning electron microscopy (SEM). The effects on FOXO3a localization were determined using an immunofluorescence assay.

### Carboxyfluorescein Diacetate Succinimidyl Ester (CFDA-SE) Cell Proliferation Assay

The CFDA SE assay was used to detect the effects of PD and Sorafenib on the proliferation of PC3 cells over time. PC3 cell suspensions were labeled with CFDA-SE, then were plated into 6-well plates overnight. The cells were then collected after being treated with the compound(s) for 7 days. The samples were analyzed for staining using a FACSCaliber flow cytometer.

### Colony Formation Assay

Cells were implanted in 6-well plates (1000 cells/well). After treatment with different concentrations of PD and Sorafenib for 10 days, the cells were fixed with paraformaldehyde and stained with crystal violet. The samples were then photographed and counted using Photoshop.

### RNA Extraction, Reverse Transcription-PCR and Real-Time Quantitative PCR

The methods used for RNA extraction, reverse transcription-PCR and real-time quantitative PCR have been described in previous articles (37). In brief, total RNA from PC3 cells was extracted using the Trizol reagent from BioFlux (Hangzhou Bioer Technology Co., Ltd., Hangzhou, China), then the RNA was reverse-transcribed. The primer sequences used for gene amplification were as follows: Akt forward, 5’-TGGTCCTGTCTTCCTCATGTTT-3’ and reverse, 5’-GCCCCTTTGACTTCTTGACC-3’; FOXO3a forward, 5’- ACGTCTTCAGGTCCTCCTGTT-3’ and reverse, 5’-GGGGAAGCACCAAAGAAGAGAG -3’; GAPDH forward, 5’- AATGGGCAGCCGTTAGGAAA-3’ and reverse, 5’-GCG CCC AAT ACG ACC AAA TC-3’. A 10 μl reaction mixture was amplified using an iQ5 machine [Bio-Rad, Hercules, CA, USA]. All amplification reactions were analyzed by the comparative threshold cycle [Ct] method and normalized to the level of GAPDH mRNA, which served as a control.

### Detection of Ubiquitinated Akt

The cells were treated with various concentrations of PD for 10 min following pretreatment with 10 µM MG132 for 6 h. Whole cell lysates were obtained by cell lysis in ice-cold RIPA buffer. After lysis, samples were shaken on a horizontal shaker for 30 min with 50% protein A/G agarose at a ratio of 100 µl per 1 ml of sample solution. After shaking, the samples were centrifuged at 14,000g at 4°C for 15 min, then the supernatant was transferred to new tubes and the protein A/G-agarose beads were discarded. At that time, 3 μl of anti-Akt antibody was added to approximately 500 μl total volume, and samples were kept at 4°C overnight. The next morning, 50% protein A/G agarose was added to the sample solution, and samples were kept at 4°Cfor 1 h. The samples were then centrifuged at 3000 r/min for 2 min. The pellet was retained and washed with pre-iced RIPA buffer 3 times for 30 min each time. The resulting pellets were kept for the subsequent Western blot analyses.

### Western Blot Analysis

Whole cell lysates were obtained by the lysis of prostate cancer cells or tumor tissue as described previously (41). The extracted proteins were subjected to SDS-PAGE according to a previously reported procedure (41). The PVDF membranes were incubated with the appropriate primary antibodies overnight at 4°C with gentle shaking. The membranes were then incubated with a goat anti-mouse/rabbit IgG horseradish peroxidase-conjugated antibody (Bio-Rad, Hercules, CA, USA). Conjugated proteins were detected using the Fusion FX5 Spectra instrument from VilberLourmat Inc. (Paris, France).

### Mouse Xenograft Models

Mouse xenograft models have been described in previous articles (40). In short, we generated PC3 cells with low expression of FOXO3a *via* the transfection of a shRNA-FOXO3a lentivirus. The resulting PC3 cells (shRNA-FOXO3a cells and MOCK (control) cells) were injected subcutaneously into the left inguinal area of nude mice (BALB/c, nu/nu, male, 6-8 weeks old). One week after tumor cell inoculation, mice bearing palpable tumors were randomly divided into control and treatment groups (6 mice/group). The PD was dissolved in the vehicle, PEG400:Saline : Ethanol (400:300:200, v/v/v), and administered (via i.p. injection) at doses of 2.5 mg/kg body weight, 5 consecutive days a week for 30 days. The control group received injections of the vehicle. The mice were sacrificed by cervical dislocation on day 30, and the tumor tissues were removed and weighed. All animals were monitored for activity, general condition, body weight, and tumor growth. The tumor size was measured every third day using calipers, and the tumor volume (cm^3^) was calculated by the following formula: (a *b^2^)/2, where ‘a’ and ‘b’ represent the longer and shorter dimensions.

### Statistical Analysis

The data for the different treatment groups are presented as the means ± standard errors. A one-way ANOVA was used to determine the significance of the effects of the treatments on the levels of apoptosis or cell death. The results were considered to be statistically significant for values of *p*< 0.05.

## Results

### PD Promotes the Anti-Tumor Effects of Sorafenib in PTEN-Deficient Prostate Cancer Cells

**Figure 1:**
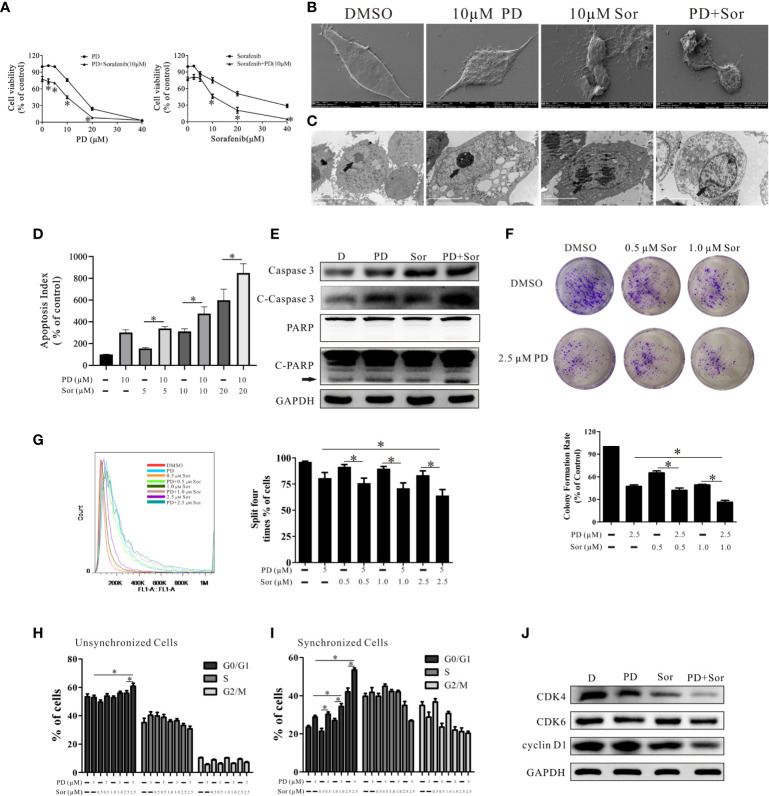
PD promotes the anti-tumor effects of sorafenib in PC3 cells. **(A)**. The effects of PD, sorafenib and PD plus sorafenib on cell viability. **(B, C)**. The changes in the cell membrane and nucleus after treatment with PD alone, sorafenib (Sor) alone or PD plus sorafenib. **(D)**. The induction of apoptosis by PD, Sor and PD + Sor. **(E)**. The protein expression levels of Caspase 3, C-Caspase 3, PARP and C-PARP were examined after cells were treated with 10 μM PD alone, 10 μM sorafenib alone or PD plus sorafenib for 6 h. **(F)**. The ability of cells to achieve colony growth was assessed after treatment with PD alone, sorafenib alone or PD plus sorafenib for 10 days. **(G)**. The proliferation of cells was monitored using the CFDA SE assay after treatment with PD alone, sorafenib alone or PD plus sorafenib for 5 days. **(H, I)**. The cell cycle distribution of PC3 cells following treatment with PD alone, sorafenib alone or PD plus sorafenib for 24 h after pre-treatment with **(H)** or without **(I)** 2 mM thymidine. **(J)**. Changes in the protein expression levels of CDK4, CDK6 and cyclin D1 after treatment with 5 μM PD alone, 2.5 μM sorafenib alone or PD plus sorafenib for 24 h. *p < 0.05.

As shown in **Figure 1A**, PD promoted the anti-tumor effects of sorafenib, and sorafenib also promoted the anti-tumor effects of PD. Of note, PD reduced the concentration of sorafenib required to reach the same level of antitumor activity by half (cell viability: 10 μM sorafenib alone vs. 5 μM sorafenib + PD, 20 μM sorafenib vs. 10 μM sorafenib + PD, 40 μM sorafenib vs 20 μM sorafenib + PD: 75.12% ± 8.53% vs. 79.17% ± 10.41%, 50.82% ± 6.94% vs. 46.14% ± 6.91%, 28.84% ± 5.37% vs. 20.72% ± 9.31%).

We also analyzed the changes in PC3 cell morphology after cells were treated with 10 μM PD alone, 10 μM sorafenib alone or PD plus sorafenib for 24 h. SEM and TEM showed that the cell membranes and nuclei were more damaged in cells treated with the combination of PD and sorafenib than in those treated with either agent alone (**Figures 1B, C**). The results of FICT and PI staining demonstrated that PD promoted sorafenib-induced apoptosis (**Figure 1D**). The combined treatment also increased the protein expression levels of apoptosis-related markers, including caspase 3, cleaved (C)-caspase 3 and C-PARP (**Figure 1E**).

In order to more extensively investigate the effects on cell proliferation, we treated cells with a non-lethal concentration for a longer time. As shown in **Figure 1F**, the combination of low concentrations of PD and sorafenib led to greater inhibition of clone formation by PC3 cells compared to treatment with sorafenib alone. However, there were only significant differences between the group treated with 1.0 μM sorafenib and 2.5 μM PD compared to the group treated with 2.5 μM PD alone. The results for CFDA SE staining were similar to those for clone formation (**Figure 1G**). Further studies of the cell cycle suggested that PD promoted sorafenib-induced cell cycle arrest at the G0/G1 phase (**Figure 1H**). The results became more pronounced when the cell cycle was synchronized prior to treatment (**Figure 1I**). We found that the protein expression of CDK4 and cyclin D1 (markers of the G0/G1 transition) was down-regulated after treatment with PD and sorafenib (**Figure 1J**). However, this increase was not observed in PTEN-positive DU145 cells (**Supplemental Figure 1**).

### PD Promotes the Anti-Tumor Effects of Sorafenib *via* an Akt-Dependent Mechanism

**Figure 2:**
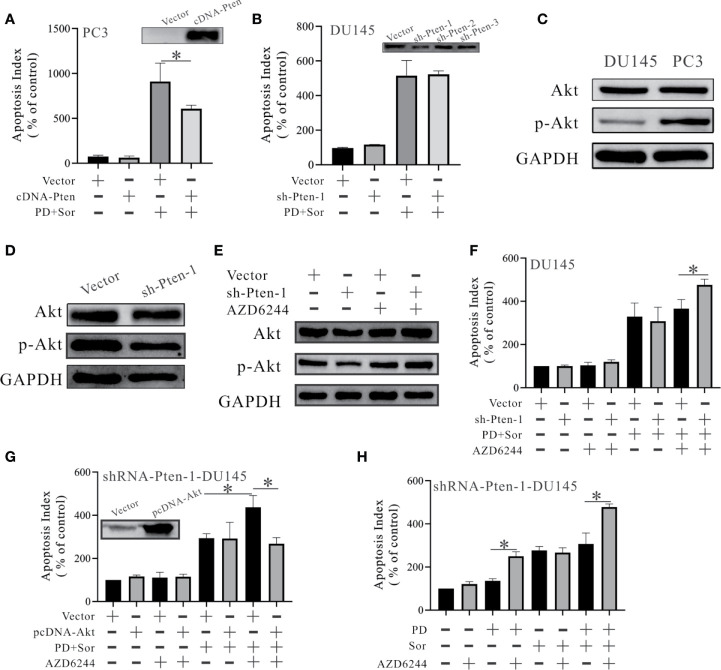
PD promotes the anti-tumor effects of sorafenib in an Akt-dependent manner in PTEN-deficient prostate cancer cells. **(A, B)** The changes in apoptosis were monitored by FITC and PI double staining in PC3 cells with PTEN overexpression **(A)** and DU145 cells with PTEN inhibition **(B)**. **(C)** The protein expression levels of Akt and p-Akt in DU145 and PC3 cells. **(D)** Changes in the protein expression levels of Akt and p-Akt in DU145 cells with PTEN inhibition. **(E)** Changes in the protein expression levels of Akt and p-Akt in DU145 cells with PTEN inhibition after pre-treatment with 50 μM AZD6244 for 2 h. **(F)** The effects of 10 μM PD combined with 10 μM sorafenib on apoptosis in shRNA-PTEN-DU145 cells with and without pre-treatment with 50 μM AZD6244 for 2 h. **(G)** Changes in the effects of PD combined with sorafenib on apoptosis in shRNA-PTEN-DU145 cells after pre-treatment with or without 50 μM AZD6244 for 2 h and with or without Akt overexpression. **(H)** Changes in the effects of 10 μM PD, 10 μM sorafenib and PD plus sorafenib on the apoptosis of shRNA-PTEN-DU145 cells after pre-treatment with or without 50 μM AZD6244 for 2 h. *p < 0.05.

To determine the role of PTEN in these observations, we knocked down and overexpressed PTEN in PTEN-positive and negative cells. The effects of combining PD with sorafenib were reduced in PC3 cells overexpressing PTEN (**Figure 2A**). However, the effects were unchanged after PTEN inhibition in DU145 cells (**Figure 2B**). PTEN functions as a classical phosphorylase that inhibits Akt activation (12). We found that Akt was activated in PC3 cells, but not DU145 cells (**Figure 2C**). Moreover, Akt wasn’t activated after PTEN inhibition in DU145 cells (**Figure 2D**). These results suggest that Akt is not important for maintaining the survival of DU145 cells. In a subsequent study, we used AZD6244 (a MEK inhibitor) to activate Akt. Treatment with AZD6244 increased the protein expression of Akt and p-Akt after PTEN inhibition (**Figure 2E**). Pre-treating DU145 cells with AZD6244 for 2 h increased the effects of the combination treatment with PD and sorafenib (**Figure 2F**). The effects of AZD6244 were abolished by Akt overexpression (**Figure 2G**). Further experiments showed that the activated Akt was inhibited by PD, but not sorafenib (**Figure 2H**).

### PD Promotes Akt Ubiquitination

**Figure 3:**
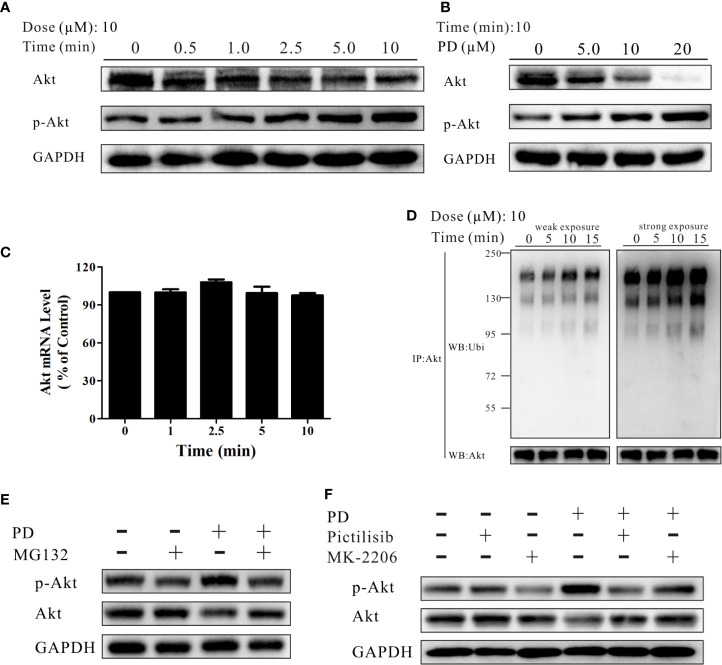
PD promotes Akt ubiquitination. **(A, B)** The changes in the protein expression levels of Akt and p-Akt induced by PD treatment were time- and concentration-dependent in PC3 cells. **(C)** The changes in the mRNA expression level of Akt after treatment with 10 μM PD. **(D)** The changes in ubiquitinated (ubi) Akt in cells following exposure to various concentrations of PD for 10 min after pre-treatment with 10 μM MG132 for 6 h (immunoprecipitation by anti-Akt, followed by Western blotting). **(E)** The changes in Akt and p-Akt in cells pre-treated with 10 μM MG132 for 6 h prior to exposure to PD for 10 min. **(F)** The changes in Akt and p-Akt in cells pre-treated with 0.5 μM pictilisib or 1.0 μM MK-2206 for 30 min prior to exposure to PD for 10 min.

Next, we explored how PD affects Akt. We found that the levels of p-Akt increased and the levels of Akt decreased at different time points after treatment with 10 µM PD (**Figure 3A**). After a 10-min incubation with PD, the levels of p-Akt increased and the levels of Akt decreased, both in a concentration-dependent manner (**Figure 3B**). These findings suggest that PD affects Akt in a time- and concentration-dependent manner.

E3 ligases bind to p-Akt, promoting its specific degradation (44). We therefore investigated whether PD inhibits Akt protein expression by affecting its mRNA expression, or whether the effects are solely due to decreased protein stability. As shown in **Figure 3C**, PD did not affect the Akt mRNA expression level. In contrast, exposure to PD resulted in a dramatic and concentration-dependent induction of Akt ubiquitination (**Figure 3D**). After treatment of PC3 cells with 10 μM MG132 for 6 h, followed by PD for 10 min, the levels of both Akt and p-Akt were strongly reduced (**Figure 3E**). To further investigate the underlying mechanism, we treated cells with pictilisib (a specific PI3K inhibitor) and MK-2206 (a specific Akt inhibitor) prior to PD treatment to confirm whether PD specifically promotes the ubiquitination of phosphorylated Akt (**Figure 3F**).

### PD Induces Cell Death Through the Akt/FOXO3a Pathway

**Figure 4:**
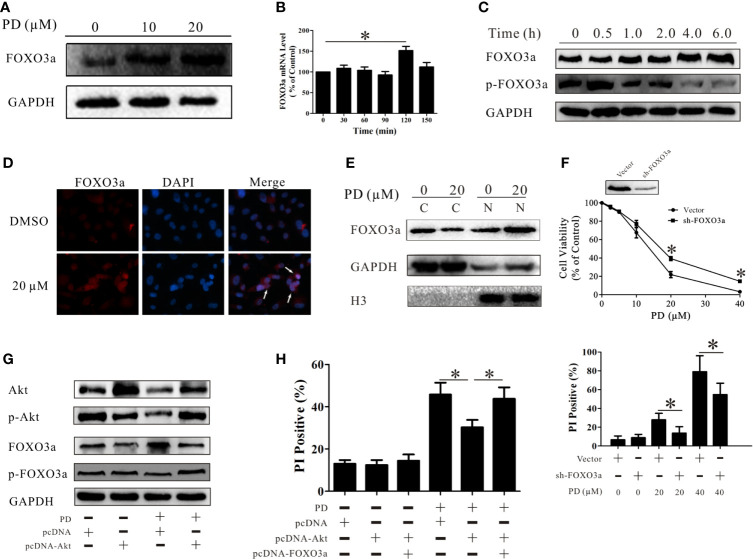
PD induces cell death through the Akt/FOXO3a pathway. **(A)** Changes in the protein expression level of FOXO3a after treatment of PC3 cells with PD. **(B)** Changes in the mRNA expression level of FOXO3a after treatment of PC3 cells with 10 μM PD for 6 h. **(C)** Changes in the protein expression levels of FOXO3a and p-FOXO3a after treatment of PC3 cells with 10 μM PD for 6h. **(D, E)** The protein expression of FOXO3a in the cytoplasm and nucleus of PC3 cells. **(F)** The effects of PD on the viability and the PI-positive rate in PC3 cells with FOXO3a inhibition. **(G)** Changes in the protein expression levels of Akt, p-Akt, FOXO3a and p-FOXO3 after the treatment of PC3 cells transfected with cDNA-Akt with 10 μM of PD for 6 h. **(H)** The PI-positive rate in cells treated with 20 μM PD for 24 h after transfection with cDNA-Akt alone or cDNA-Akt plus cDNA-FOXO3a. *p < 0.05.

FOXO3a plays an important role in the development of prostate cancer and is a downstream molecule of Akt (25). We observed that PD increased the protein expression level of FOXO3a in PC3 cells (**Figure 4A**). Exposure to PD resulted in the induction of FOXO3a mRNA expression at 120 min (**Figure 4B**), and the induction of FOXO3a and inhibition of p-FOXO3a protein expression in a time-dependent manner (**Figure 4C**).

It has been demonstrated that FOXO3a controls the rate of transcription of genetic information from DNA to messenger RNA by binding to a specific DNA sequence in the nucleus (45). Immunofluorescence assays and Western blot analyses showed that PD treatment increased the protein expression levels of FOXO3a in the nuclei in PC3 cells (**Figures 4D, E**). This suggests that PD promotes the function of FOXO3a in these cells. To examine the role of FOXO3a in the effects of PD, we suppressed FOXO3a expression in PC3 cells. The effects of PD on cell death were decreased in the absence of FOXO3a (**Figure 4F**). Additionally, these effects of FOXO3a were reversed after AKT overexpression (**Figure 4G**). As shown in **Figure 4H**, the induction of cell death by PD was decreased after Akt overexpression, and this was reversed after simultaneous over-expression of AKT and FOXO3a.

### PD Regulates the Downstream Targets of FOXO3a

**Figure 5:**
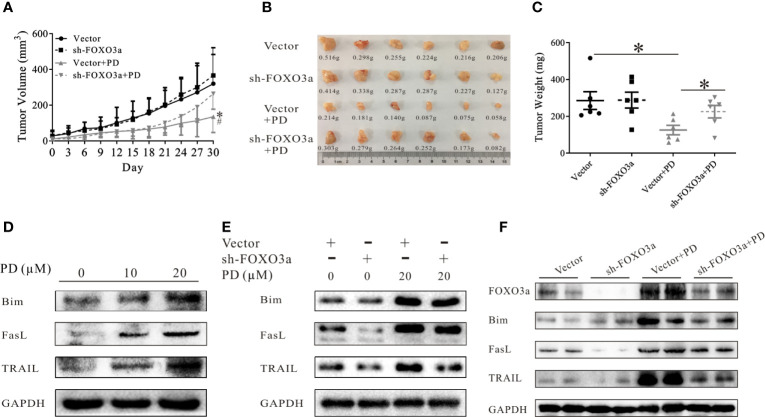
PD regulates the downstream targets of FOXO3a. **(A–C)**. The effects of PD on the growth of xenograft PC3 tumors after FOXO3a inhibition. **(D)**. The changes in the expression of FOXO3a target proteins (Bim, Fasl and TRAIL) in PC3 cells were monitored by Western blotting. **(E, F)**, The effects of PD on FOXO3a target protein expression *in vitro* **(E)** and *in vivo* **(F)** after FOXO3a inhibition. *p < 0.05.

We then confirmed the role of FOXO3a in an *in vivo* model. As shown in **Figures 5A–C**, the anti-tumor effects of PD were reduced after the expression of FOXO3a was suppressed *in vivo*. Bim, Fasl and TRAIL are downstream genes of FOXO3a (31–33, 46). PD increased the protein expression levels of all three of these in PC3 cells (**Figure 5D**). These effects were decreased when the expression of FOXO3a was suppressed (**Figure 5E**). The same results were obtained *in vivo* (**Figure 5F**). It is worth noting that TRAIL expression was more strongly affected than Bim and Fasl expression (**Figures 5E, F**). This suggests that PD affects TRAIL through the Akt/FOXO3a pathway.

### PD Promotes the Anti-Tumor Effects of Sorafenib by Increasing the Activity and Expression of FOXO3a

**Figure 6:**
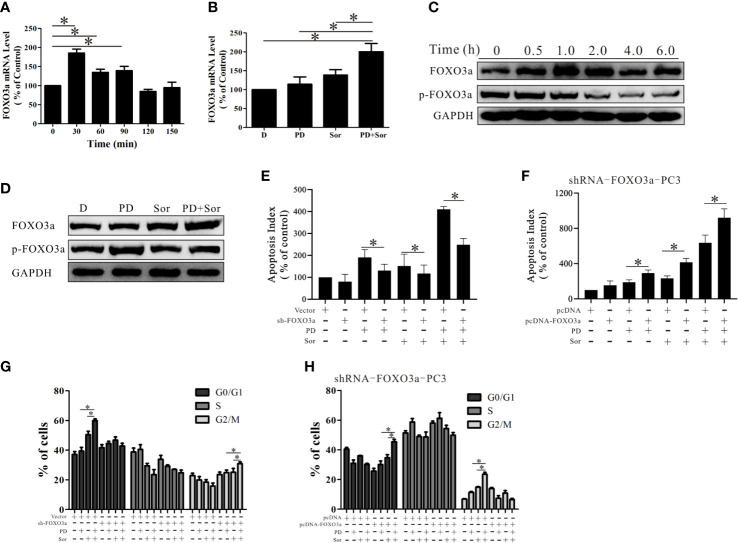
PD promotes the anti-tumor effects of sorafenib by increasing the activity and expression of FOXO3a. **(A)** The mRNA expression level of FOXO3a after treatment of PC3 cells with 10 μM PD plus 10 μM sorafenib. **(B)** The mRNA expression level of FOXO3a after treatment with 10 μM PD alone, 10 μM sorafenib alone or PD plus sorafenib for 30 min. **(C)** The protein expression levels of FOXO3a and p-FOXO3a after treatment with 10 μM PD plus 10 μM sorafenib. **(D)** The protein expression levels of FOXO3a and p-FOXO3a after treatment with 10 μM PD alone, 10 μM sorafenib alone or PD plus sorafenib for 30 min. **(E)** The effects of PD, sorafenib or PD plus sorafenib on early apoptosis in cells with FOXO3a inhibition. **(F)** The changes in apoptosis of shRNA-FOXO3a PC3 cells with cDNA-FOXO3a treated with PD, sorafenib or PD plus sorafenib. **(G)** The effects of PD, sorafenib or PD plus sorafenib on the cell cycle distribution after FOXO3a inhibition and pre-treatment with 2 mM thymidine for 24 h. **(H)** The effects of PD, sorafenib or PD plus sorafenib on the cell cycle distribution of shRNA-FOXO3a PC3 cells after transfection with cDNA-FOXO3a for 6 h and pre-treatment with 2 mM thymidine for 24 h. *p < 0.05.

The above results demonstrate that PD induces cell death through the Akt/FOXO3a pathway in PTEN-deficient prostate cancer. However, the role of FOXO3a in the effects of combining PD with sorafenib was not clear. As shown in **Figure 6A**, the mRNA expression of FOXO3a was increased 30 min to 90 min after treatment with the combination of PD and sorafenib. Importantly, the FOXO3a mRNA level was not significantly increased after treatment with PD or sorafenib alone for 30 min (**Figure 6B**). The results for the FOXO3a protein level were similar to those for the mRNA level (**Figures 6C, D**). These results suggest that the combination of PD and sorafenib promoted FOXO3a expression more than treatment with PD or sorafenib alone.

When FOXO3a expression was reduced by shRNA treatment, the effects of the combination treatment were reduced (**Figure 6E**). The efficacy of the combination was restored when additional FOXO3a was expressed (**Figure 6F**). Further studies of the cell cycle suggested that PD promoted sorafenib-induced cell cycle arrest at the G0/G1 phase (**Figure 6G**). After FOXO3a expression was decreased, the combined effects of PD and sorafenib on G0/G1 arrest were abolished, but there was a slight but significant induction of G2/M arrest (**Figure 6G**). This effect was reversed by the restoration of FOXO3a expression (**Figure 6H**). These findings suggest that FOXO3a promotes the anti-tumor effects of the combination treatment with sorafenib and PD.

## Discussion

Combining different treatments is a common method to improve drug efficacy and reduce side effects. Previous studies showed that combination treatment with an AR inhibitor (enzalutamide), antibiotic (clofoctol) or a natural product (Nobiletin) promoted the anti-cancer effects of sorafenib in castration-resistant prostate cancer (47–49). These studies mainly observed the effect and mechanism of drug combination on prostate cancer, but did not discuss the limitations of these strategies. We herein defined a novel combination strategy to treat androgen-independent and PTEN-deficient prostate cancer. Our findings demonstrated that PD enhanced the anti-cancer effects of sorafenib in this type of prostate cancer (**Figure 1**). A drawback to this combination is that PTEN deficiency is not the only condition required for PD to promote the effects of sorafenib- the Akt pathway also needs to be a key pathway maintaining the survival of these cancer cells (**Figure 2**). PTEN, as a dephosphorylase of Akt, inhibits Akt activation and limits Akt function. While PTEN deficiency is a pre-condition for Akt activation, the absence of PTEN does not mean that there is Akt activation. Several studies have shown that PTEN might have additional functions that are independent of the PI3K-Akt pathway (50). Hence, it is necessary to pay attention to the status of both PTEN and Akt when considering the combination of PD or other Akt inhibitors with sorafenib. In addition, the effects of the combination were reversed by over-expressing Akt in the cells. Thus, it appears that PD only enhances the anti-cancer effects of sorafenib in androgen-independent, PTEN-deficient and Akt-active prostate cancer cells.

Studies have shown that the progression to CRPC after ADT is closely associated with the interaction of the PI3K complex with the AR pathway (51). In fact, when an Akt inhibitor was combined with an AR inhibitor, there was superior antitumor activity compared to treatment with the AR inhibitor alone in patients with PTEN-deficient tumors (14). An Akt inhibitor (AZD8186) also promoted the anti-cancer effects of docetaxel (a cytotoxic drug) against PTEN-deficient prostate cancer (52). Clinical studies have also confirmed that there is a more potent response in prostate cancer patients with PTEN deficiency when they were treated with docetaxel combined with AZD8186 (53). Previous research indicated that PD inhibits tumor cells migration, invasion, and growth, and induces autophagy *via* suppression of Akt-associated pathways (54–57). However, all of these studies examined the long-term effects of PD on AKT. The present study focused on the short-term effects of PD on the protein expression of p-Akt and Akt, and found an increase in p-Akt and decrease in the other forms (**Figures 3A, B**). Further results showed that PD increased Akt ubiquitination (**Figure 3D**). Interestingly, pre-treatment of prostate cancer cells with pictilisib (a PI3K-specific inhibitor) or MK-2206 (a p-Akt-specific inhibitor) reversed this decrease in Akt protein expression (**Figure 3E**). A previous study indicated that ubiquitin ligases preferentially bind to p-Akt (Thr308 and Ser473) to promote the specific degradation of activated Akt (44). Thus, PD may have induced Akt ubiquitination by promoting its phosphorylation, which occurs within a short period of time after treatment.

FOXO3a is expressed at a lower level in PTEN-negative cells than in PTEN-positive prostate cancer cells (58). Li et al. showed that miR-26a promotes prostate cancer development through the PTEN/Akt/FOXO3a pathway (59). Triphenyltin carboxylate targets the PTEN/Akt/FOXO3a pathway to inhibit the growth of prostate cancer with PTEN deficiency (60). Our present data demonstrated that PD promotes both the protein expression and function of FOXO3a in PTEN-negative cells (**Figure 4**). We also observed that the effects of PD on PC3 cells were reversed after inhibition of FOXO3a expression (**Figure 4F**). However, Akt phosphorylates FOXO3a to induce its ubiquitination (61). As shown in **Figure 4H**, the effects of PD on FOXO3a were reversed when cDNA for Akt was transfected into prostate cancer cells. The effects were abolished after co-transfection of cDNA-Akt and cDNA-FOXO3a (**Figure 4G**), indicating that PD induced cell death through the Akt/FOXO3a pathway. Of note, PD activated p-Akt and increased p-FOXO3a after 30 min, but the level of t-FOXO3a did not decrease (**Figure 4C**), and the mRNA level of FOXO3a was not increased at that time point (**Figure 4B**). This means that FOXO3 is phosphorylated, but not further ubiquitinated, following treatment. Our previous research demonstrated that PD inhibits MDM2 (a FOXO3a ubiquitin ligase) (37), further suggesting that PD exerts its effects by altering the ubiquitination of its targets.

It is well-established that genes encoding cell death-related proteins, such as FasL, TRAIL and Bim, are regulated by FOXO3a (62–64). Of note, PD promoted the protein expression of FasL, TRAIL and Bim induced by FOXO3a, with TRAIL exhibiting the most pronounced changes (**Figures 5E, F**). Previous studies demonstrated that TRAIL promoted the antitumor effects of sorafenib (65). Hence, PD can promote the anti-tumor effects of sorafenib by promoting the protein expression of TRAIL. Yang et al. previously showed that FOXO3a induced 5’ AR promoter activity *via* binding to a consensus DNA-binding sequence in the AR 5’ promoter (-1290 to -1297; 5’-TTGTTTCA-3’) (66). Under these conditions, the AR played important protective roles to prevent cell death (66). Therefore, the status of the AR should be considered when using FOXO3a agonists to inhibit prostate cancer.

Research by Yamamoto and colleagues showed that a single dose of sorafenib (30-60 mg/kg) decreased the activation of the PI3K/AKT/mTOR signaling axis in a mouse model of PTEN-deficient prostate cancer (20). We showed that the antitumor effects of sorafenib were decreased after FOXO3a expression was inhibited (**Figure 6E**), but were unchangedafter Akt expression was activated (**Figure 2H**). This result suggested that sorafenib inhibited prostate cancer *via* a mechanism dependent on FOXO3a, and that it promoted FOXO3a function through pathways involving molecules other than Akt. This may be one of the reasons why sorafenib promoted the effects of an AR inhibitor in AR-dependent prostate cancer. However, FOXO3a has been shown to react differently to sorafenib in different cell types. For example, sorafenib up-regulated the protein expression of FOXO3a in B- and T-lymphoblastic cells (67) and down-regulated the expression in liver cancer cells (68). Thus, the effects are complex, and likely cell context-specific.

In summary, our present findings provide evidence that PD increases the ubiquitination of Akt. PD also promotes the protein and mRNA expression of FOXO3a. We demonstrated that FOXO3a-mediated effects appear to play an important role in PD-induced prostate cancer cell death. Moreover, the increase in FOXO3a function caused by PD promoted the anti-tumor effects of sorafenib. Therefore, PD (alone or in combination with sorafenib) can be used for the treatment of PTEN-deficient and androgen-independent prostate cancer. Further studies will be needed to elucidate the detailed mechanisms underlying these findings. However, the current results provide support for further studies of PD as a candidate natural product-based preventive, auxiliary and/or therapeutic agent for prostate cancer.

## Data Availability Statement

The original contributions presented in the study are included in the article/**Supplementary Material**. Further inquiries can be directed to the corresponding author.

## Ethics Statement

The animal study was reviewed and approved by Laboratory Animal Welfare and Ethics Committee of the Third Military Medical University.

## Author Contributions

HX and ZL designed research. ZL, WS, YWZ, and CPW performed research and wrote the paper. MZ, HW, and YZ analyzed data. MZ contributed the statistical analysis. NL contributed the reagents. All authors contributed to the article and approved the submitted version.

## Funding

This work was supported by grants from the National Natural Science Foundation of China (Nos. 81603347), the Natural Science Foundation of Chongqing (cstc2020jcyj-msxmX0499), and a Basic Science and Frontier Technology Research Project of Chongqing (CSTC2017jcyjAX0305).

## Conflict of Interest

The authors declare that the research was conducted in the absence of any commercial or financial relationships that could be construed as a potential conflict of interest.

## Supplementary Material

Supplementary Figure 1 PD does not promote the anti-tumor effects of sorafenib in PTEN-positive prostate cancer. **(A)** The effects of PD, sorafenib and PD plus sorafenib on cell viability. **(B)** The changes in apoptosis were monitored by FITC and PI double staining. **(C)** The proliferation of cells was monitored using the CFDA SE assay after treatment with PD alone, sorafenib alone or PD plus sorafenib for 5 days.
