# Supplementary figures and images for: Combined Anti-Cancer Effects of Platycodin D and Sorafenib on Androgen-Independent and PTEN-Deficient Prostate Cancer
Source: Front Oncol. 2021 May 7;11:648985. doi: 10.3389/fonc.2021.648985 (PMC8138035; doi:10.3389/fonc.2021.648985)

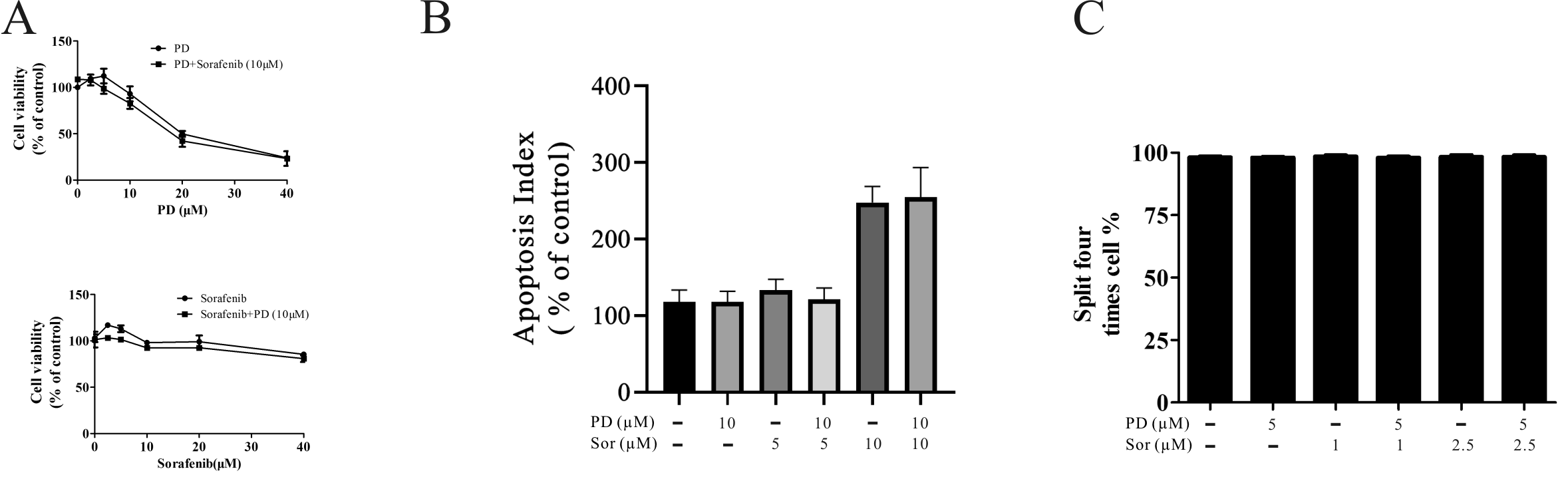

Supplement: Supplementary Figure 1 — PD does not promote the anti-tumor effects of sorafenib in PTEN-positive prostate cancer. (A) The effects of PD, sorafenib and PD plus sorafenib on cell viability. (B) The changes in apoptosis were monitored by FITC and PI double staining. (C) The proliferation of cells was monitored using the CFDA SE assay after treatment with PD alone, sorafenib alone or PD plus sorafenib for 5 days. [file Image_1.tif]
